# Case of Enteritis, Accompanied with a Preter-Natural Formation of the Ileum

**Published:** 1816-06

**Authors:** John W. Francis

**Affiliations:** New-York.


*For the London Medical and Physical Journal.*

### *Case of Enteritis, accompanied with a Preter-natural Formation of the Ileum;* by John W. Francis, M. D. of New-York.

THE writer of the following paper was an eye-witness to most of the facts which he relates. They are taken from memoranda made at the request of his preceptor, Dr. David Hosack, in whose practice the case occurred.

On the morning of December the 22d, 1809, Dr. Hosack was requested to visit a Captain D——, aged about thirty-five, of a slender habit of body, who was represented to be in an alarming condition. At the first view of the patient, it was perceived that he was afflicted with all the symptoms characteristic of enteritis, accompanied with those of ileus, viz. an acute and constant pain in the whole abdominal region, particularly about the umbilicus; the abdomen greatly distended, hard, and extremely sensible to the slightest touch, or whenever he attempted to move; vomiting of stercoraceous matter, and constipated state of the bowels; pulses small, tense, and frequent; respiration hurried and anxious; countenance livid; heat of the body increased somewhat beyond its natural temperature; and excessive thirst. These symptoms were attended with a great prostration of strength, and an extreme degree of restlessness.

Upon

Upon inquiring into the history of his complaint, it appeared that he had been first attacked while at the theatre, on Wednesday evening, the 20th. On the morning of the day following, he was visited by an eminent physician, who directed an antispasmodic mixture, the symptoms of his disease being, at that time, but slight. Deriving no relief from the medicine prescribed, Dr. Hosack was called upon on Friday morning, the 22d, between the hours of eight and nine, when he found him labouring under all the symptoms above described.

From the best information that could be obtained, it was rendered highly probable, that the exciting cause of his complaint was *cold.* He had repeatedly been subjected to attacks of this kind, though less violent than the present, for several years past; at which times he was relieved by the ordinary method of treatment.

Immediate recourse was now had to the lancet, and he lost blood to the amount of eighteen ounces. A cathartic, composed of the pulv. jalap. and sub-muriate of quicksilver, each ten grains, was directed to be given, which was rejected in about an hour after he had taken it; and a similar one repeated with the same result. Blisters were applied near the umbilicus; fomentations of vinegar and water over the whole abdomen; and enemata of the oleum ricini and tinct. assafœtid. were administered. These were partly discharged by vomiting, which afforded abundant proof that an inverted action of the whole intestinal canal had already taken place. In the afternoon, the several applications to his surface were repeated; and, during the remainder of the day, he took, in divided doses, no less than two scruples of the sub-muriate of quicksilver, combined with opium and camphor, which, however, were rejected by vomiting shortly after they were taken. The enemata, rendered more active, were again given, but with no advantage. At this time Dr. Miller visited the patient, in conjunction with Dr. Post and Dr. Hosack. They united in recommending a continuance of the same mode of treatment that had been pursued. In this condition he passed the night, the constipation of the bowels obstinately resisting every means used to obtain an evacuation.

On the morning of the 23d, the sub-muriate of quicksilver, combined with opium, was again directed, in doses of fifteen grains every two hours. The warm bath was at the same time employed. It produced a temporary mitigation of his symptoms, but left him still more enfeebled. His fate, which for some time had been probable, now became almost certain. The vomiting, which, within the first thirty hours from the commencement of his disease, had become stercoraceous, and which had continued, with but little intermission, to the present time, was now renewed. Attempts were made to allay it by the free use of the tincture of opium and other remedies usually indicated under similar circumstances. The effect was an aggravation of all the symptoms. At 10 o'clock, P. M. his dissolution was momentarily expected. His pulses were scarcely perceptible, and his extremities cold.

He expired on Sunday morning, the 24th, at six o'clock, the vomiting having been incessant until about twenty minutes before his death.

*Morbid Appearances on Dissection.*—At two o'clock in the afternoon, the body was examined, in the presence of the attending physicians, and several other professional characters. The abdomen was tense, and greatly distended: upon making a longitudinal incision into it, a considerable quantity of serous fluid issued out. Having completed the division, the intestines were found in a highly-inflamed state, and of a dark-red colour; the peritoneum lining the abdomen was also much inflamed, and covered with coagulable lymph. A remarkable deviation from the ordinary structure of the parts was now discovered to exist: a portion of intestine, attaching itself to the umbilicus, formed a union between it and a part of the intestinal canal. Upon further examination, this appendix was observed to be a *diverticulum* from the ileum. At the place of its union with the ileum it was enlarged and inflamed, in common with the upper portion of the small intestines; the remaining part was of a natural colour, and so intimately connected by its blind extremity at the umbilicus, as to leave little doubt of its being an original malformation. The ileum, above this appendix, was very much inflated, extremely vascular, and, in size, equal to the transverse colon; while the lower portion was greatly contracted, and twisted round the diverticulum; and in this manner had been the means at least of aggravating, if not of inducing, the inflammation, and its consequences, in this particular part of the intestinal canal. This portion of the ileum was of a dark livid appearance, and had lost its natural tenacity. The great intestines were found completely emptied of their contents, and preternaturally contracted in their diameters throughout their course.

The omentum, transverse colon, and stomach, were, at first, altogether concealed, by the distended state of the small intestines, and found in close contact with the diaphragm. The omentum was irregularly drawn together. No unnatural appearance of the transverse colon was remarked.

The

The stomach lay in a circumscribed situation, was not more than two inches in width, and contracted in the same proportion throughout its whole extent. It was entirely empty: upon a minute inspection, no discolouration or affection of its coats was seen.

